# High-frequency burst spiking in layer 5 thick-tufted pyramids of rat primary somatosensory cortex encodes exploratory touch

**DOI:** 10.1038/s42003-021-02241-8

**Published:** 2021-06-10

**Authors:** Christiaan P. J. de Kock, Jean Pie, Anton W. Pieneman, Rebecca A. Mease, Arco Bast, Jason M. Guest, Marcel Oberlaender, Huibert D. Mansvelder, Bert Sakmann

**Affiliations:** 1 Department of Integrative Neurophysiology, Center for Neurogenomics and Cognitive Research, Neuroscience Campus Amsterdam, VU, Amsterdam, the Netherlands; 2 University of Amsterdam, Swammerdam Institute for Life Sciences, Amsterdam, Netherlands; 3 Institute of Physiology and Pathophysiology, Heidelberg University, Heidelberg, Germany; 4 Max Planck Group: In Silico Brain Sciences, Center of Advanced European Studies and Research, Bonn, Germany; 5 Max Planck Institute for Neurobiology, Martinsried, Germany

**Keywords:** Sensory processing, Whisker system

## Abstract

Diversity of cell-types that collectively shape the cortical microcircuit ensures the necessary computational richness to orchestrate a wide variety of behaviors. The information content embedded in spiking activity of identified cell-types remain unclear to a large extent. Here, we recorded spike responses upon whisker touch of anatomically identified excitatory cell-types in primary somatosensory cortex in naive, untrained rats. We find major differences across layers and cell-types. The temporal structure of spontaneous spiking contains high-frequency bursts (≥100 Hz) in all morphological cell-types but a significant increase upon whisker touch is restricted to layer L5 thick-tufted pyramids (L5tts) and thus provides a distinct neurophysiological signature. We find that whisker touch can also be decoded from L5tt bursting, but not from other cell-types. We observed high-frequency bursts in L5tts projecting to different subcortical regions, including thalamus, midbrain and brainstem. We conclude that bursts in L5tts allow accurate coding and decoding of exploratory whisker touch.

## Abstract (web summary)

In order to investigate the information encoded by spiking activity in different neuronal cell types in the primary somatosensory cortex, de Kock et al performed electrophysiological recordings in untrained rats. They demonstrated that an increase in high-frequency burst spiking in thick tufted pyramids in layer 5 of the cortex allow accurate encoding of exploratory whisker touch.

## Introduction

The cortical circuitry consists of cell types that are classified by molecular, structural, and functional signatures^1,2^. A wealth of data characterizing the organizational rules of cortical connections have implied the existence of a canonical microcircuit, referred to as the “cortical column”^3^. The stereotypic wiring diagram includes in- and output layers, containing a collection of heterogeneous inhibitory and excitatory cell types^4^. Computational performance by individual cell types is ideally studied under conditions of natural behavior but the technical challenge of stable electrophysiological or optical recordings in awake behaving animals is typically achieved at the expense of cell-type identification. In the context of understanding how the cortical column triggers behaviors, the cellular identity of recorded neurons across cortical layers is however critical, including layer 5 (L5), which is the main output layer of the cortical column^5^.

L(ayer) 5 consists of an intermingled population of two major output neurons: L5 intratelencephalic (IT) and L5 pyramidal tract (PT) neurons, respectively^1^, commonly referred to as L5 slender-tufted (L5st) and L5 thick-tufted (L5tt) pyramids. These two L5 output cell types differ in key structural and functional properties^6–8^, including afferent input sources^9^ and output projection targets^8,10,11^.

In visual cortex, L5st and L5tt pyramids represent specialized output channels^12^ and show cell-type-specific sensitivity to stimulus orientation and direction, temporal frequency tuning curves, and binocularity index^13,14^. In the prefrontal and motor cortex, L5st and L5tt populations form discrete, highly selective microcircuits^15,16^ and contribute distinctly to working memory processes^17^. Also in auditory and somatosensory cortices, functional properties correlate to L5st versus L5tt identity^8,18,19^. Collectively, a consistent organizational principle emerges across cortical areas in which L5st and L5tt (i.e., L5 IT vs L5 PT) pyramidal populations act as distinct functional entities that contribute highly specialized computational properties to the cortical column^6,20,21^.

The rodent somatosensory (barrel) column has been widely studied to uncover the contribution of individual cell types to neural processing underlying whisker-guided decision-making, including that of L5 output neurons^22^. L5st pyramids receive dense input from the posterior medial thalamic nucleus (POm), are part of the paralemniscal pathway, their efferent projections target striatum and S1 in the contralateral hemisphere and do not respond reliably to passive touch^9,23,24^. L5tt pyramids receive multiple segregated inputs (including POm and VPM), are part of the lemniscal pathway, project to multiple downstream targets, and reliably respond to passive whisker touch^11,24,25^. L5tts exhibit coincidence-detection capability when segregated inputs are simultaneously active^26^, leading to dendritic Ca2+-dependent plateau potentials and ultimately perception^8^. The dendritic potentials are expected to induce somatic action potential (AP) burst spiking and this burst spiking is central to theories of the cortical neural code^27,28^ and was recently shown to control learning^29^. In addition, burst spiking may be critical to overcome synaptic depression in long-range anatomical projections, which is particularly relevant for the L5tt-to-POm pathway^30,31^. To aid functional interpretations of their morphological properties and uncover the contribution of L5st and L5tt pyramids to sensory-guided behavior, the cell-type-specific spiking activity needs to be disentangled in awake animals.

Behaving rodents deploy a highly stereotypical active tactile behavior, resulting in efficient, brief haptic windows of 20–65 ms, which mimics a hand grasp in human^32^. Head-fixed rodents can adapt their whisking strategy^33^, but create comparable temporal windows during whisker-based decision-making tasks to extract salient information^34^. To measure spiking of identified neurons in naturally behaving animals, head-fixation can be readily combined with single-cell recordings for dye-labeling and post hoc neuronal reconstruction^35,36^. To uncover the spiking profile within a cortical column and cell-type-specific representation of naive tactile sensation (i.e., active object touch), we recorded from anatomically identified neurons in head-fixed rats that were habituated to head-fixation but otherwise untrained and naive to the sensory conditions during the recording. We find that exploratory whisker touch evokes high-frequency burst spiking almost exclusively in (a subset of) L5tt pyramids and these high-frequency burst events carry information on whisker touch not present in spike counts.

## Results

**Fig. 1:**
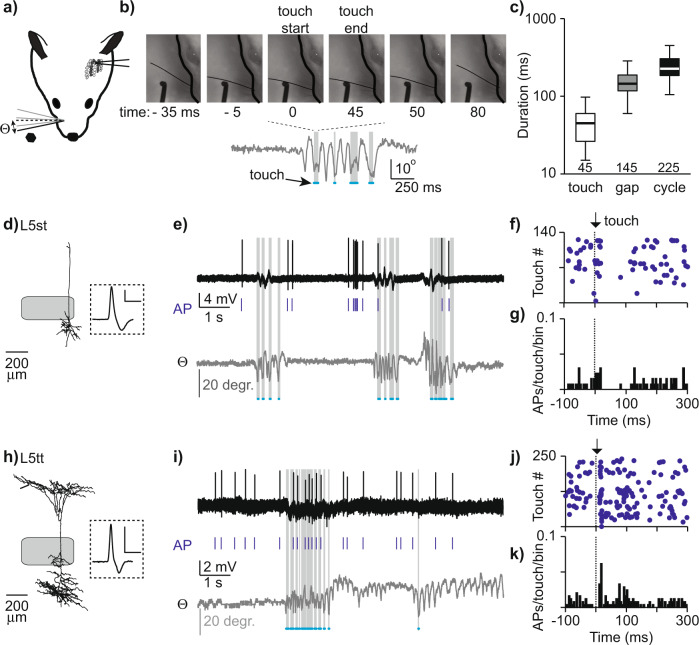
Loose-patch recordings in primary somatosensory cortex during naive whisker touch. **a** Cartoon of recording configuration with loose-patch, single-unit recording in primary somatosensory cortex in combination with high-speed videography of self-induced whisker use and object touch. **b** Series of single frames from high-speed videography to illustrate offline analysis of whisker position (Θ) and object touch at 5 ms resolution. **c** Population statistics for duration of a whisker touch, the gap between touch end and consecutive touch start, and cycle, which is the time between consecutive touch starts. Boxplots show median as central mark, the edges of the box the 25th and 75th percentiles, the whiskers extend to the most extreme data points. Outliers are omitted for clarity.  **d** Neurolucida reconstruction of an example L5 slender-tufted pyramid (L5st) and average (regular) AP waveform. Scale bar inset: 4 mV, 1 ms. **e** Loose-patch recording of the morphologically identified L5st showing the continuous voltage trace (black), extracted APs (ticks), and offline tracked whisker position (gray) with object touches highlighted by blue bars and gray shading. **f** Raster plot with AP spiking aligned to consecutive touches. **g** Peri-stimulus-time-histogram (PSTH) aligned to touch start. **h**–**k** Analogous to **d**–**g** but for L5 thick-tufted pyramid (L5tt). Note the cell-type-specific spiking response upon whisker touch (**g** versus **k**).

To study layer- and cell-type-specific representation of untrained, exploratory whisker touches in the primary somatosensory cortex of awake rats, loose-patch (single-unit) recordings were combined with high-speed videography of whisker position (frame rate 200/s, Fig. 1). From the whisking behavior, we quantified episodes of quiescence, active whisking, and object touch as well as the temporal sequence of touch events (Fig. 1b). Across the population of *n* = 80 recordings (8460 touches), median touch duration was 45 ms (1st/3rd quartile 27–60 ms, Fig. 1c), the gap between touch end and subsequent touch start 145 ms (1st/3rd quartile 119–186 ms) and time between consecutive touch starts (i.e., cycle) was 225 ms (1st/3rd quartile 185–305 ms). Cycle time thus translates to a dominant frequency of 4.4 Hz for subsequent touch events (1st/3rd quartile 3.3–5.4 Hz). The touch duration in habituated, head-fixed awake rats is indistinguishable from behavioral characteristics of tactile exploration in freely moving rats^37^ and approaches the theoretical optimized window of tactile exploration of objects^32^, thus suggesting that whisking under awake head-fixed conditions resembles natural whisking behavior.

Action potential (AP) spiking by single units was recorded and neurons were labeled with biocytin for post hoc morphological reconstruction and cell-type identification. We determined AP responses to whisker touch for morphologically defined cell types, exemplified by a representative L(ayer) 5 slender-tufted (5st) and a representative L5 thick-tufted (5tt) pyramid, respectively (Fig. 1). Data included morphological reconstructions (Figs. 1d, h), loose-patch recordings in combination with offline whisker tracking (Figs. 1e, i), raster plots to align APs to individual touch events (Figs. 1f, j), and peri-stimulus time histograms to compute the average response rate across touch events (APs/touch, Figs. 1g, k). Touch did not increase spiking activity in this L5st example (Fig. 1g), contrasting the L5tt example, which showed a clear increase in spiking activity upon touch (albeit at low average number of APs per individual touch, Fig. 1k).

**Fig. 2:**
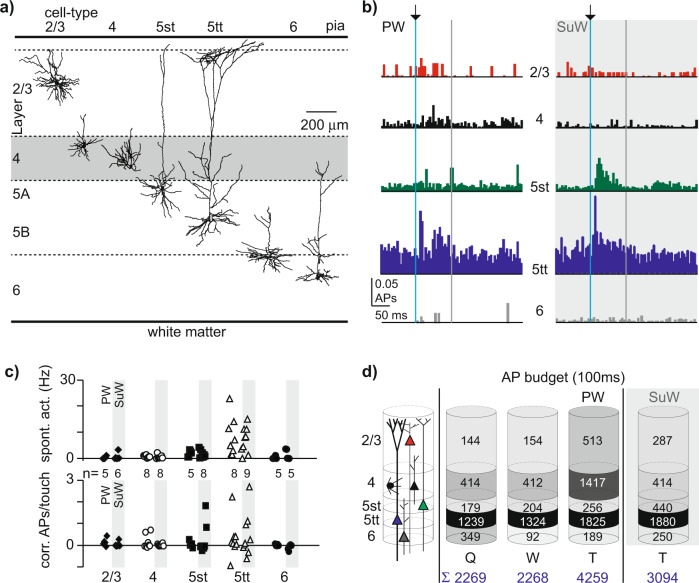
Single-cell and population spiking upon naive object touch. **a** Representative morphologies of cell types across S1 layers. In L(ayer 4), data from pyramidal neurons and spiny stellates is pooled. In L6, data from corticocortical and corticothalamic neurons are pooled. In L5, slender-tufted (st) and thick-tufted (tt) pyramids represent distinct cell types. **b** Population PSTHs for five major excitatory cell types triggered to onset of PW touch (PW) or surround whisker touch (SuW), normalized for the number of touch events for individual recordings. Bin size 5 ms, blue line indicates touch start, the gray line end of 0–100 ms touch start window, respectively. The L5st-SuW population PSTH was dominated by a single L5st. Colors distinguish cell types and match the color code in **d**. For PW/SuW: L2/3 *n* = 5/*n* = 6, L4 *n* = 8/*n* = 8, L5st = 5/*n* = 8, L5tt *n* = 8/*n* = 9, L6 *n* = 5/*n* = 5. **c** Cell-type-specific spiking activity (in Hz) during quiescence (top) and upon touch (below, corrected for spontaneous activity). For n-numbers PW/SuW: analogous to data in Fig. 2b. **d** AP budget for 0–100 ms windows across behavioral states (quiescent, whisking, and touch) and PW touch versus SuW touch. Gray shading is normalized to the cell-type-specific maximal number of APs within a behavioral condition.

To obtain an estimate of spike output of the cortical column, we quantified touch-induced spiking across layers and identified cell types. The spike output is also indicative of the information broadcasted within and between columns and to subcortical targets. Recordings were included based on regular spiking waveform (*n* = 67) and were grouped according to principal (PW, *n* = 31) or surround (SuW, *n* = 36) whisker touch depending on anatomical location (confirmed by post hoc histology). Next, recordings were sub-classified into five excitatory cell types (L2/3, L4, L5st, L5tt, and L6, Fig. 2a), based on morphology, layer-location, or cell-type-specific functional signature (Methods).

We used the spectrogram function (Matlab, Mathworks) to define episodes of quiescence (Q) and whisking (W). Touch events were extracted through frame-by-frame inspection of the high-speed video. We aligned spiking exclusively to touch start and did not include additional variables such as whisker angle, velocity, acceleration, period, and curvature (Fig. 2b). Spontaneous spiking activity during quiescence was cell-type specific^38,39^ and only a subset of neurons across layers responded to active touch (PW, 61%, SuW 39%, *p* = 0.09, Fisher exact, two-sided). Population averages were thus dominated by a subset of recordings^39^. After correction for spontaneous activity, population responses upon touch were sparse across all cell types (<1 AP/touch, Fig. 2c, Table 1). Finally, we observed a larger fraction of short-latency responses in PW columns compared with SuW columns (39% vs 14%, *p* = 0.03, Fisher exact, two-sided).

**Table 1:** State-dependent spiking frequencies.

Cell-type	Quiescent	Whisking	PW touch	Norm. PW touch	SuW touch	Norm. SuW touch
L2/3	0.53 ± 1.00 (11)	0.57 ± 0.99 (11)	1.90 ± 2.16 (5)	1.54 ± 1.80 (5)	1.06 ± 2.51 (6)	0.39 ± 1.19 (6)
L4	0.72 ± 0.62 (16)	0.71 ± 0.89 (16)	2.45 ± 3.02 (8)	1.62 ± 3.14 (8)	0.72 ± 0.98 (8)	0.12 ± 0.52 (8)
L5st	1.80 ± 1.35 (13)	2.04 ± 2.08 (13)	2.56 ± 1.51 (5)	0.62 ± 1.38 (5)	4.41 ± 6.92 (8)	2.70 ± 7.09 (8)
L5tt	7.68 ± 6.09 (17)	8.21 ± 6.96 (16)	11.32 ± 10.36 (8)	2.94 ± 8.87 (8)	11.65 ± 13.59 (9)	4.59 ± 10.05 (9)
L6	0.90 ± 1.47 (10)	0.24 ± 0.33 (9)	0.49 ± 0.74 (5)	0.18 ± 0.39 (5)	0.64 ± 0.86 (5)	−0.84 ± 1.30 (5)

Cell-type-specific spiking rates (in APs/s) and touch-evoked spiking rates corrected for spiking rates during Quiescent episodes (“norm.”, in APs/s). Note that spiking rates are calculated for principal (PW) and surround whisker (SuW) touch separately. Values represent mean ± st. dev. and number of observations (*n*=).

Next, we generated cell-type-specific AP budgets across behavioral states by multiplying cell-type-specific touch responses (APs/touch/unit) with total counts of each cell-type per column^40^. We find that under quiescent conditions (Q), the ensemble of 14,800 regular spiking units in a single column generates 2300 APs (in a 100 ms time window, Fig. 2d). Upon principal whisker touch (PW-T), the AP budget for regular spiking units across layers increased to 4300 APs (0–100 ms after touch start). This increase was significantly higher compared to the increase to 3100 APs after SuW touch (SuW-T, Mann–Whitney *p* < 0.05).

In addition, the AP budget revealed that relative increases upon touch (from Q to PW-T) were largest for L4 cells in PW columns, owing to large cell counts for L4 (5800) neurons^40^. L5st neurons showed only a moderate increase during whisking, but these episodes constituted only small whisker movements since larger movements (free whisking in air) resulted in object touch. L5tt cells had the highest absolute spiking frequency and L5tt-specific AP budgets were comparable for PW and SuW touches (Fig. 2d, Mann–Whitney, *p* > 0.05). This resembles broad receptive field (RF) properties reported for this cell-type under anesthesia^24,41^.

**Fig. 3:**
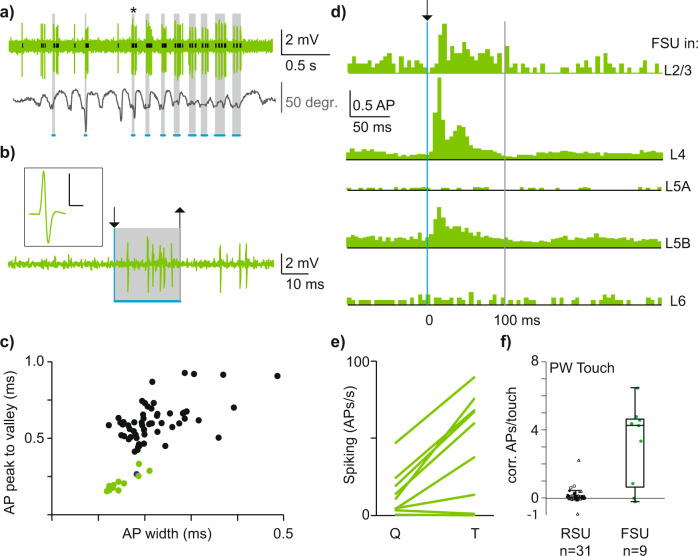
Dense representation of naive object touch in fast-spiking units across layers. **a** Loose-patch recording of an individual fast-spiking-unit (FSU) in L4 including whisker position and naive object touch. Note robust increase in spiking upon naive object touch. **b** A single touch from **a** (with asterisk) at increased temporal resolution. The absolute touch duration is indicated with gray shading. Arrows indicate touch start and touch end, respectively. Scale bars inset: 0.5 mV, 0.5 ms. **c** Average waveform parameters for RSUs and FSUs across layers of PW and SuW column. FSUs in green, RSUs in black. One AP waveform (blue bullet) clustered with FSUs but morphological reconstruction revealed L5st identity and the unit was classified according to morphology. **d** Individual PSTHs for *n* = 5 example FSUs recorded across layers. **e** Spiking rate for PW-FSUs across behavioral states (Q = quiescent, T = touch). **f** Sparse versus dense coding of touch for regular versus fast-spiking units, respectively (Mann–Whitney, *p* < 0.01). Boxplots show median as central mark, the edges of the box the 25th and 75th percentiles, the whiskers extend to the most extreme data points, and the outliers are plotted individually.

In cortical microcircuits, a tight balance exists between excitation and inhibition^42^, which is influenced by intracortical and thalamocortical inputs^36,43^. As we found low spiking rates across excitatory cell types, the next aim was to quantify naive touch-evoked responses in fast-spiking (putative inhibitory) neurons. Without visual control for targeted loose-patch recordings, however, or genetic control of inhibitory cell types existing for mice^36^, the ~80/20 ratio of excitatory/inhibitory cell counts makes it challenging to record from inhibitory neurons, which is further biased towards excitatory neurons by their relatively larger soma size. To overcome these methodological challenges, we used small tip, high resistance pipettes to target fast-spiking units (FSUs) across layers of principal whisker columns (PW *n* = 9). A subset of these FSUs was morphologically identified, but this did not result in unambiguous sub-classification and single-cell data were thus pooled. We found short-latency, robust increases of spiking for a subset of FSUs across layers (Fig. 3a–d), which were identified based on AP waveform (Fig. 3c)^44^. This resembled the pronounced thalamocortical drive during somatosensation recently shown for L4 FSUs^36^. Spiking rates of individual FSUs during naive PW touch exceeded spiking rates of RSUs across layers (Figs. 3e, f, median RSU: 0.06 APs/PW touch 1st/3rd quartile: −0.02–0.16, median FSU: 4.27 APs/PW Touch, 1st/3rd quartile: 0.86–4.58, (Mann–Whitney, *p* < 0.01)).

### High-frequency burst spiking

**Fig. 4:**
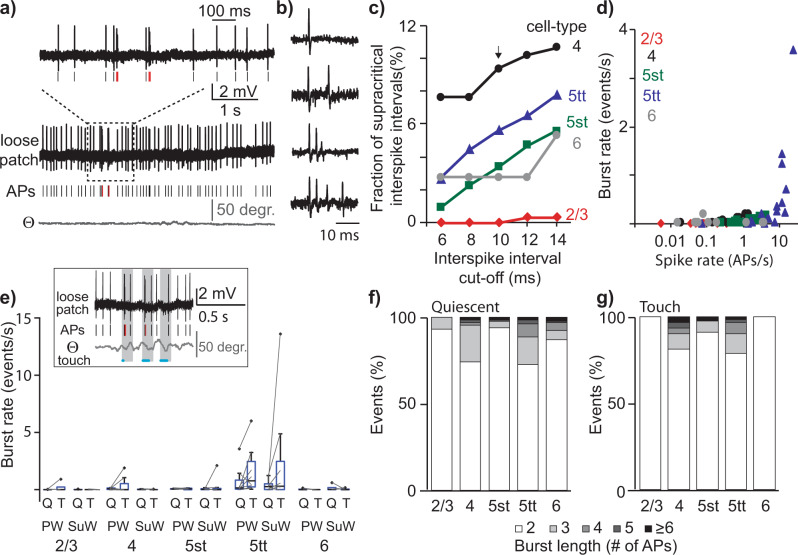
Cell-type-specific high-frequency bursting. **a** Spiking activity of an example L5tt recording during quiescent episodes with single spikes indicated by black ticks and spikes at supracritical frequency (≥100 Hz) by red ticks, respectively. **b** Example traces of a single spike (upper trace) and burst events of 2–4 consecutive spikes at supracritical frequency (≥100 Hz). **c** The median fraction of high-frequency bursts detected during spontaneous spiking activity (quiescent episodes) depends on the cutoff criteria (1st–3rd quartile range omitted for clarity). Arrow indicates the cutoff criterion used in subsequent analyses. **d** Scatterplot illustrating spike rate (APs/s) of individual recordings during quiescent episodes and burst rate (events/s) (Pearson correlation, Rho 0.66, *p* < 0.0001). **e** Burst rate (events/s) during quiescent (Q) and touch (T) windows, respectively for different excitatory cell types. Boxplots show median as central mark, the edges of the box the 25th and 75th percentiles, the whiskers extend to the most extreme data points, and the outliers are plotted individually. **f** Burst length as the number of action potentials (APs), relative to the total number of events observed during quiescent windows. **g** Analogous to (f) , but for Touch windows.

**Fig. 5:**
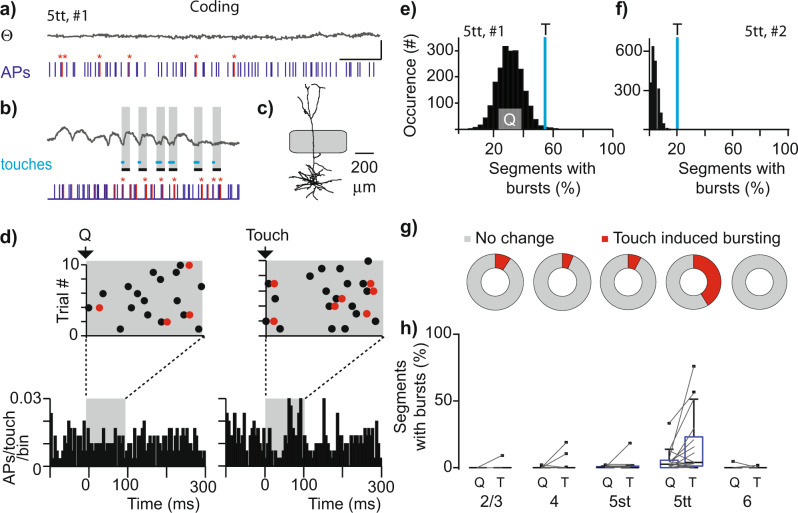
Coding of touch using high-frequency AP bursts (≥100 Hz). **a** Loose-patch recording of a single L5tt illustrating high-frequency burst spiking during Quiescent episodes. Action potentials (APs) of instantaneous frequency ≥100 Hz are highlighted with red asterisks. Θ denotes whisker position. Scale bar: 25 degrees, 500 ms. **b** Analogous to (a) but for episode with consecutive touches. Gray shaded boxes indicate 100 ms after touch onset. **c** Digital reconstruction reveals L5tt morphology. **d** Raster plots and PSTHs triggered to random quiescent and true touch times. APs at instantaneous frequency of ≥100 Hz are indicated by red bullets. **e** Histogram of the bootstrapped burst distribution based on 100 ms Quiescent segments and the recorded fraction of touches (0–100 ms touch start window) with high-frequency bursts as a blue line. Note that fraction of touches with bursts exceed 99th percentile of distribution, equivalent of *p* < 0.01. **f** Bootstrapped burst distribution for 2nd L5tt example, *p* < 0.01. **g** Seven out of 17 L5tt pyramids show an increase of bursting upon touch, which is a significantly higher fraction compared with additional excitatory cell types (3/50), *p* < 0.01, two-sided Chi-square test). **h** Fraction of segments with a burst in Q(uiescent) and T(touch) windows as a function of cell-type (touch: Kruskal–Wallis, *p* < 0.0001, post hoc L5tt vs L2/3 or L4 or L6, all *p* < 0.001). Boxplots show median as central mark, the edges of the box the 25th and 75th percentiles, the whiskers extend to the most extreme data points, and the outliers are plotted individually.

The AP budget (Fig. 2) provides an estimate for the number of spikes that represent a whisker touch (i.e., the rate code), which does not take into consideration information that could be included through exact spike timing. To determine whether the temporal characteristics of spike trains contribute to sensory processing, we quantified the occurrence of high-frequency burst events (Fig.  4). These high-frequency bursts are defined by a critical frequency^45^ and bursts in excitatory cell types are thought to increase information content and facilitate dendritic integration through dendritic back propagation^27^. Bursts may be necessary to overcome synaptic depression and relay salient sensory information from cortex to subcortical regions^30^. Finally, high-frequency bursts contribute to sensory perception and control learning^8,29^. Burst events are subsequently defined as a set of spikes with consecutive interspike intervals (ISI) of ≤10 ms. During quiescent episodes, high-frequency bursts were observed in 52 out of 67 recordings (78%) including all morphological cell types (Figs. 4a, c, a single AP and events of different lengths exemplified in Fig. 4b). The occurrence of supracritical burst spiking depends on the cutoff frequency (Fig. 4c) and based on previous, detailed characterization of the critical frequency for L5tts^45^, we set the threshold for burst detection at instantaneous spiking ≥100 Hz (or ISI of ≤10 ms). During Quiescent behavior, burst rate (events/s) significantly correlates to spike rate (APs/s, Fig. 4d, Pearson’s correlation, Rho 0.66, *p* < 0.0001) and burst spiking was particularly prominent in L5tts. Upon exploratory whisker touch, burst rate increased for individual cells but only reached significance for the population of L5tts (burst rate quiescent vs touch, Fig. 5e, Wilcoxon paired-test, *p* = 0.02, *n* = 17). Burst events were short and consisted typically of 2–5 APs, both during Quiescent (Fig. 4f) and Touch (Fig. 4g) windows. To conclude, whisker touch-evoked burst spiking, particularly in L5tt neurons, and burst events typically consisted of 2–5 APs with ISI ≤ 10 ms.

The increased burst rate during touch could indicate that touches are consistently represented by burst events. Alternatively, a minor fraction of touches coincides with multiple bursts. To uncover the coding strategy, we next quantified high-frequency burst spiking during individual touches and illustrate our strategy for a representative L5tt recording (Fig. 5a–d). This example recording showed AP spiking at 22.9 AP/s during quiescent episodes and 24.3 APs/s during the 30 touch events recorded (0–100 ms post-touch onset window). To determine whether burst occurrence during individual touches exceeded burst occurrence during spontaneous conditions, we first randomly sampled 30 windows of 100 ms during Quiescence (matching 30 touch events) and determined the fraction of these windows that contained at least one high-frequency burst. Next, we sampled a different random 30 windows and repeated this procedure a total of 2000 times. The median fraction of quiescent segments containing at least one burst was 33.3% (Fig. 5e). The fraction of 30 touches associated with a burst was 56.7% (0–100 ms post-touch window). This value maps beyond the 99th percentile of the chance of bursts during quiescent windows, indicating a significant increase of bursting upon touch (*p* < 0.01). A second L5tt example showed that 4.2% of quiescent windows associated with bursts, which increased to 20.8% upon touch (48 touches, *p* < 0.01, Fig. 5f). On the population level, we found that high-frequency bursts correlated to evoked spike rate (Pearson correlation, *p* < 0.001, *n* = 67), but only a minor fraction of individual recordings showed a statistically significant increase in bursting upon touch (10 out 57, 18%, Fig. 5g). The neurons with increased bursting upon touch were predominantly L5tts (*n* = 7 out of 17) with only incidental contributions by other excitatory neurons across the S1 column (*n* = 1/11 L2/3; *n* = 1/16 L4; *n* = 1/13 L5st; *n* = 0/10 L6). The likelihood of touch-induced bursting in L5tts (7 out of 17) was significantly higher compared to the additional excitatory cell types in the S1 column (3 out of 50, *p* < 0.01, two-sided Chi-square test). Thus, even though bursting during quiescent episodes is observed across all morphological cell types (Fig. 4c), the representation of naive whisker touch by burst events is observed almost exclusively in (a subset of) L5tts (Fig. 5h).

### Decoding touch events from spiking rates and burst events

**Fig. 6:**
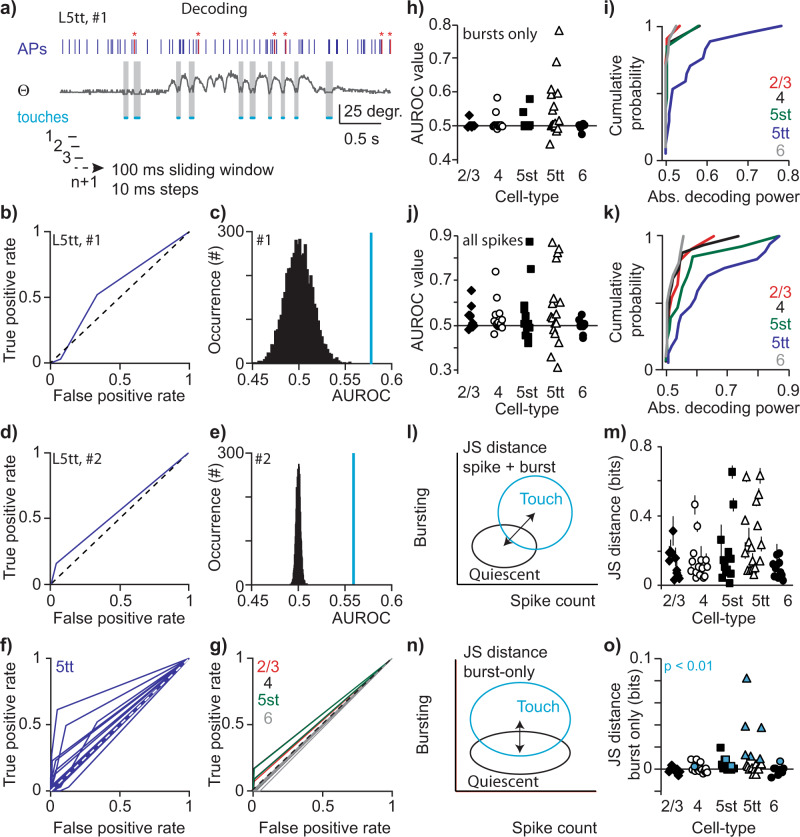
Decoding of touch using high-frequency AP burst events. **a** Example trace of same L5tt neuron of Fig. 5a–e with 100 ms sliding window (+10 ms steps) to obtain spiking characteristics and associated behavior. High-frequency bursts are highlighted in red, accompanied by red asterisks. Decoding was analyzed by a 100 ms sliding window (10 ms steps) to sample the rate of bursts. **b** Receiver operating characteristic curve (ROC-curve), decoding performance was estimated as the area under the ROC curve (AUROC). Bracket line indicates chance level. **c** Histogram of AUROC values after shuffling the data for 5000 repetitions. Note that the true AUROC value (blue line) is beyond the 99th percentile of the shuffled distribution, indicating significant decoding power. Example matches Fig. 5, 5tt, #1). **d**, **e** Analogous to **b**, **c** but for example L5tt, #2, matching Fig. 5, 5tt, #2). Note that the true AUROC value exceeds the 99th percentile of the shuffled distribution, indicating significant decoding power. **f** ROCs for the population of L5tts. **g** ROCs for individual L2/3 (in red), L4 (black), L5st (green), and L6 (gray) recordings. Bracket line indicates chance level. **h** AUROC values as a function of cell type. **i** Cumulative distribution of absolute AUROC value as a function of cell-type (Kruskal–Wallis, *p* < 0.01, post hoc test L5tt vs L2/3 < 0.01, L5tt vs L6 *p* < 0.05, Kolmogorov–Smirnov test for comparison of two distributions, L5tt vs rest, *p* < 0.01). **j** AUROC values as a function of cell type, computed on decoding value of spike rate. **k** Cumulative distribution of absolute AUROC value as a function of cell-type, computed on decoding value of spike rate (Kruskal–Wallis, *p* < 0.01, post hoc test L5tt vs L2/3 < 0.01, L5tt vs L6 *p* < 0.05, Kolmogorov–Smirnov test for comparison of two distributions, L5tt vs rest, *p* < 0.01). **l** Cartoon illustrating Jensen–Shannon metric (JS distance, see Methods). **m** JS distance including 95% confidence intervals (error bars) for individual identified neurons, based on spike count and bursting. **n** Cartoon illustrating JS metric for “burst only”. **o** JS distance for “burst only” (correcting √JSD “spike count + bursting” with √JSD for “spike only”) is cell-type specific (Kruskal–Wallis, *p* < 0.001) and significantly higher for L5tt compared with L2/3, L4, and L6 (Dunn’s Multiple comparisons test, *p* < 0.05). Blue markers represent neurons with significant information in bursts not present in spike counts (*p* < 0.01 after correction for false discovery rate, Benjamini–Hochberg procedure, see Methods).

To understand how sensory representation may be internally decoded, “reverse” analyses are equally necessary. The next goal was therefore to test whether an observer can infer behavior (i.e., occurrence of a whisker touch) from rate and/or temporal codes in morphologically identified neurons. We set the sliding window to 100 ms (step size 10 ms) on single-cell spiking data (Fig. 6a). We obtained the spike count and number of burst events for all windows with a touch (Touch distribution) versus all windows without a touch (Quiescent distribution, see Methods). Windows from Whisking episodes or 100 ms windows with mixed behavior were not included. We built a receiver operating characteristic curve (ROC-curve) by varying the classification criterion along with all burst event values and computed the true-positive rate (the proportion of 100 ms windows with a touch and classified as touch) and the false-positive rate (the proportion of 100 ms windows without touch, yet classified as touch). Finally, the decoding performance was calculated as the area under the ROC-curve (AUROC). This is first exemplified for the L5tt examples illustrated during the coding analysis. The first neuron showed a significant increase in high-frequency bursts upon touch (Fig. 5e) but also a relatively high likelihood of burst events during Quiescent episodes (Q, 33.3%). In the reverse analysis, we found that the true-positive rate still exceeded the false-positive rate and decoding power (AUROC value) was significantly higher compared with chance (the same data but in which we shuffled the touch labels) (Fig. 6b, c, *p* < 0.01). Similarly, the L5tt with low likelihood of spontaneous high-frequency bursts and significant increase of burst spiking upon touch (Fig. 5f, 5tt #2) also showed significant decoding power relative to shuffled data (Fig. 6d, e). This indicates that for these two example recordings, high-frequency burst events provide information on whether a touch occurred.

In the L5tt population, we found several ROC curves significantly deviating from the diagonal and decoding power thus above chance (Fig. 6f). This was different for the additional excitatory cell types in the S1 column (i.e., L2/3, L4, L5st, L6, Fig. 6g) as most AUROC values were close to chance level (i.e., 0.5, Fig. 6h). This is in line with the lack of evoked burst spiking upon whisker touch (Fig. 5g, h). Absolute decoding power of high-frequency burst events was cell-type specific (Kruskal–Wallis, *p* < 0.01) and decoding power was significantly higher in L5tts compared with L2/3, L4, L5st, or L6 cell types (Fig. 6i, 2-sample Kolmogorov–Smirnov, L5tt vs rest, *p* < 0.0001).

We repeated the decoding (AUROC) analysis for spike count and found that significant decoding power was distributed more evenly across excitatory cell types, in line with community consensus that S1 cell types increase spiking rate upon whisker stimulation (Fig. 6j). For a subset of recordings, we found decoding power in decreased spiking activity (L2/3: 2 out of 11, L4: 2 out of 16, L5st: 5 out of 13, L5tts: 4 out of 17, L6: 2 out of 10), confirming heterogeneity of cortical spiking upon stimulus processing^8,39,46^. We again found that absolute decoding power of spike count (increase or decrease) was cell-type specific (Kruskal–Wallis *p* < 0.01) and significantly higher in L5tts compared with L2/3, L4, L5st, or L6 cell types (Figs. 6k, two-sample Kolmogorov–Smirnov, L5tt vs rest, *p* < 0.05). Since evoked spike rate and occurrence of burst events are co-dependent measures, the decoding power of burst events in L5tt could be a consequence of the correlation with spike rate.

To quantify whether burst events contain information on touch occurrence, which is not already carried in spike counts, we used information theory. The Jensen–Shannon divergence (JSD) quantifies the similarity between probability distributions and its square root (√JSD, Methods) provides a true distance metric^47^ (Fig. 6l). First, for every neuron, we used the √JSD to quantify the separability between touch and quiescent conditions based on both spike count and burst event presence. We found that the √JSD was highest for L5tt (median L5tt 0.22, range additional excitatory cell types 0.08–0.14) and was cell-type specific (Fig. 6m, Kruskal–Wallis, *p* < 0.05). We then shuffled the labels for sliding windows on whether a burst event occurred or not, thus removing the information present in burst events. The resulting “spike rate only” √JSD allowed us to calculate the difference between the √JSD for “spike rate only” and the √JSD for “spike rate + bursting”. This provided the √JSD measure for “burst only” (Fig. 6n) and represented the separability of touch and quiescent conditions based exclusively on burst event presence, independent from spike rate (Fig. 6o). We found that the √JSD for “burst only” was again highest for L5tt (Kruskal–Wallis, *p* < 0.001) and significantly higher for L5tt compared with L2/3, L4, and L6 (Dunn’s Multiple comparisons test, *p* < 0.05). Finally, 6 out of 17 individual L5tts showed significant information in the √JSD measure for bursting, which is a higher fraction compared with additional excitatory cell types in the S1 column (5 out of 50, two-sided Chi-square *p* < 0.05). This last step indicates that *n* = 6 L5tts encode touch-related information through burst events in addition to that already present in spike count.

To determine the robustness of our conclusions, we removed the single L5tt recording from our data set that showed the highest spike rate upon touch (2.7 APs/touch, SuW, Fig. 2c) and redid all major analyses. We consistently confirmed statistical significance across coding, decoding, and information theory analyses. This includes increased burst rate upon touch for L5tts (Fig. 4e, Wilcoxon-matched pairs, *n* = 16, *p* < 0.05), coding of touches by bursting in almost exclusively (a subset of) L5tts (Fig. 5g, Chi-square, *n* = 66, *p* < 0.01, Fig. 5h, Wilcoxon matches pairs, *n* = 16, *p* < 0.05), cell-type-specific decoding of bursts and decoding of spikes (Fig. 6h–k, Kruskal–Wallis and Kolmogorov–Smirnov, *n* = 66, all *p* < 0.05), Jensen–Shannon distance on “spike + burst” (Fig. 6m, Kruskal–Wallis, *n* = 66, *p* < 0.05) and Jensen–Shannon distance on “burst only” (Fig. 6o, Kruskal–Wallis, *n* = 66, *p* < 0.01).

To summarize, our combination of analyses illustrates that high-frequency burst events allow encoding and decoding touch events but this neurophysiological correlate is highly specific to L5tts.

**Fig. 7:**
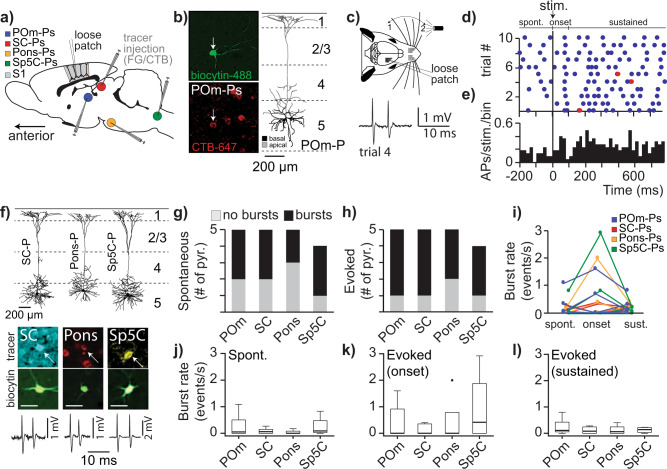
High-frequency bursting upon whisker stimulation is observed in L5tts projecting to different subcortical targets. **a** Injections of retrograde tracers into the major subcortical targets of L5tts in rat primary somatosensory cortex (S1), allowing back-labeling and projection-specific identification. Panel adapted from ref. ^11^. **b** Loose-patch recording and biocytin labeling leads to identification of cell-type-specific morphology and projection target. Here, biocytin labeling of exemplary neuron in L5, after retrograde tracer injections into the SC (FG), Pons (CTB-594), and POm (CTB-647). Slices were stained with Alexa-405 and Alexa-488 to reveal retrogradely and biocytin-labeled neurons (white arrow), respectively. Right panel: 3D morphological reconstruction of the exemplary L5tt. **c** Cartoon of recording configuration with loose-patch, single-unit recording in primary somatosensory cortex in combination with passive, multi-whisker stimulation (air-puff). Example voltage trace illustrates a stimulus-evoked high-frequency burst of the POm-projecting L5tt pyramid shown in Fig. 7b. **d** Raster plot with AP spiking aligned to consecutive touches. APs at instantaneous frequency of ≥100 Hz are indicated by red bullets. **e** Peri-stimulus-time-histogram (PSTH) aligned to touch start. **f** Examples of L5tt reconstructions projecting to different subcortical targets and high-frequency bursts (bottom trace) observed in these neurons. Scale bar biocytin-filled cell bodies: 50 μm. **g**, **h** High-frequency bursts are observed during **g** spontaneous and **h** stimulus-evoked windows in L5tts with different project-targets (individual ratios not significant relative to general population, Chi-Square *p* > 0.05). **i** Burst rate for individual L5tt neurons across conditions with color code representing projecting target (legend in **a**). **j**–**l** Fraction of supracritical burst events upon whisker stimulus is generalizable across L5tts with different projection targets (Kruskal–Wallis, *p* > 0.05) as shown for spontaneous spiking (**j**), the onset of evoked activity (**k**), and in the window representing sustained activity (**l**), respectively. Boxplots show median as central mark, the edges of the box the 25th and 75th percentiles, the whiskers extend to the most extreme data points, and the outliers are plotted individually.

The population of L5tts constitute the major output of the S1 column and consist of neurons projecting selectively to one of the subcortical targets including POm, superior colliculus (SC), Sp5C (caudalis subnucleus of the spinal trigeminal complex), or Pons^8,10,11^. In particular, the projection from S1 L5tts to POm has been characterized in detail revealing that—under in vivo conditions—the L5tt-POm synaptic connection is highly depressed^31,48,49^. Simulations indicated that high-frequency burst spiking in L5tts can overcome this tonic synaptic depression and induce POm spiking^30^. In addition, dendritic Ca^2+^ plateau potentials in L5tts projecting to POm and SC support sensory-guided perception^8^, which may implicate that high-frequency spike bursting in these L5tts not only supports local cellular computation but is also important for long-range output and eventually for behavior. To validate whether high-frequency bursting occurs in selective or multiple L5tt pathways, we quantified bursting upon whisker stimulation in morphologically identified L5tts from which subcortical targets were also revealed through post hoc histology (Fig. 7a, b,^11^). In short, different retrograde tracers were injected into three targets of the same animal. Retrograde injections were combined with in vivo cell-attached recordings and whisker stimulation (Fig. 7c–e). Each recorded neuron was labeled with biocytin to allow post hoc identification and reconstruction of dendritic morphology (Fig. 7f). We recovered 19 L5tts for which projection target could be unambiguously confirmed; of this population, 11 illustrated high-frequency bursts during spontaneous spiking activity (58%), confirming that the majority of L5tts are capable of high-frequency bursting during anesthesia (88.9%)^50^, or quiescence episodes in awake rats (94%, Fig. 4). Most importantly, we found high-frequency bursts in all sub-categories based on projection target, including POm-projecting L5tts (Fig. 7g, for spontaneous and Fig. 7h for evoked conditions, respectively) with no significant differences between sub-categories (Chi-Square, *p* > 0.05). Upon robust, multi-whisker stimulation (using air-puff), a subset of L5tts responded with short-latency burst events, with individual L5tts distributed over projection-specific populations (Fig. 7i). Also, high-frequency burst rate (events/s) was comparable across projection-specific sub-categories, both for spontaneous activity (Fig. 7j), response onset upon whisker stimulation (Fig. 7k), and the response window representing sustained spiking activity (Fig. 7l, Kruskal–Wallis, *p* = 0.82).

In view of the challenging nature of these experiments, all statistical tests were performed on a limited number of observations. Based on 54 identified L5tt recordings across experimental settings, however, we conclude that (1) high-frequency burst spiking is a general phenomenon across experimental conditions, (2) bursts allow robust coding and reliable decoding of naive touch in a subset of L5tts, (3) bursts upon whisker stimulation are observed in L5tts projecting to POm, SC, Pons, and Sp5C, thus representing a neuronal signature for whisker touch conveyed along multiple downstream projection pathways.

## Discussion

Cortical layers across sensory, motor, associative, and prefrontal areas contain mixed populations of excitatory neurons, including two functional and morphological pyramidal subtypes in layer 5^1,6,15^. The L5tt/PT neurons and L5st/IT neurons differ in dendritic and axonal morphology, projection targets, wiring properties within the cortical circuitry, and spiking characteristics. Thus, L5st/IT and L5tt/PT neurons contribute differently to behavior^8,12,51^.

Also in primary somatosensory cortex (S1), L5st and L5tt have different structural properties, and spiking characteristics during behavioral performance are cell-type specific. Properties of L5 output neurons, however, are reported without further cell-type sub-categorization^39,52^. Here, we used loose-patch recordings in awake behaving animals in combination with biocytin labeling and post hoc histology to study cell-type-specific representation of whisker touch. We show that exploratory touch in untrained, naive rats (i) is represented by a layer- and cell-type specific (sparse) code in excitatory neurons of S1 (Fig. 2), (ii) leads to robust activity in a subset of FSUs (putative inhibitory neurons, Fig. 3), (iii) significantly increases high-frequency burst spiking in L5tts (but not any other excitatory cell-type, Fig. 4, Fig. 5), (iv) decoding power to infer touches from bursts is highest in L5tts compared with other excitatory cell types and burst events carry information not present in spike count only (Fig. 6) and finally, (v) high-frequency bursts are observed in L5tts with different subcortical projection targets (Fig. 7).

Our data thus show that the spiking activity of the two main output cell types in L5 is highly cell-type specific; L5tts but not L5sts encode whisker touch through high-frequency bursts (Figs. 1, 2, 5). This dichotomy adds to the functional differentiation we observed during free whisking in naive rats, which predominantly leads to increased activity in L5sts from L5A^38^.

It may be surprising that L5sts have a low spike response probability after PW touch, as these neurons can show increased activity during free whisking^38^. L5tt neurons do not generally show increased spiking rates during whisking but a subset responds robustly to active touch. One possible explanation for sparse response rates is that we recorded spiking rates using a limited stimulus parameter space (a simple object touched during protraction). We can therefore not exclude the possibility that response rates for individual neurons may be maximized for retraction-induced touch or tuned to touches in dorsal vs ventral angular direction. Alternatively, only a small fraction of neurons across layers is involved in coding haptic events^39^, which may be better sampled with dense probe recordings^39,53^. It is tempting, however, to speculate that the response dichotomy between the two long-range projecting cell types in rat S1-L5 suggests that whisking and touch-specific signals could be conveyed to these two cell types via anatomically separate pathways^54,55^. This may represent an organizational principle that emerges at the level of the cortical circuitry, as previous studies on para- and lemniscal pathways in the trigemo-thalamo-cortical pathway in mice indicate that whisking and touch are not segregated at the level of thalamic nuclei^56,57^.

An additional important result from quantifying touch responses and the comparison between L5st and L5tt cell types is the increase in the occurrence of high-frequency spike bursts. These burst events are recorded at the soma, but have been shown to result from dendritic activity^58,59^, which in turn modulates conscious perception^8,60,61^. We observed increased burst spiking in a subset of L5tts after both PW- and SuW touch, which is different relative to any other excitatory cell type (Fig. 5). This increased bursting specific to L5tts could reflect the integration of different input pathways, leading to coincidence detection and dendritic electrogenesis^58^.

The increase of spike bursts by L5tts in response to whisker touch could be an efficient strategy to signal that a touch occurred to downstream targets of S1. This is particularly relevant for the L5tt-POm synapse where burst spiking is an effective strategy to overcome tonic synaptic depression and recruit the thalamo-cortico-thalamic loop during active touch^27,28,48,49^.

It remains to be determined how the information pathways on whisker motion and whisker touch are merged to form a comprehensive sensory percept^62^. The integration could occur in S1 since silencing of S1 leads to a dramatic drop in behavioral performance of mice that learned to report the position of an object^63^. The working hypothesis that emerged is that a distributed and weak signal on whisker position (in the azimuth plane) allows strong modulation of neuronal activity upon touch (reviewed in ref. ^64^). The cellular correlate of this hypothesis remains to be revealed though, partly due to methodological challenges when electrical recordings in behaving animals have to be combined with single-cell identification. Here, we put forward the idea that L5tts represent the most likely site of integration, since (1) these neurons receive information from L5sts, (2) potentially receive information from both VPM and POm projections, (3) a subset shows increased activity during whisker motion, (4) L5tts are modulated by whisker phase^38^ and (5) L5tts encode whisker touch (Figs. 1, 2) and object position^61^. Collectively, L5tt output could reflect information on both whisker position and touch^65^.

Spiking in L5tts is typically highly variable, in that a large range of spontaneous and sensory-evoked spiking rates has been reported^11,24^. Accurate decoding of individual spikes and bursts for single L5tts during active somatosensation will be a challenging task, complicated by the assumption that rate and temporal codes (single spikes and high-frequency bursts, respectively) will almost certainly carry information on touch duration, object texture/shape, whisker curvature, phase, angle, velocity etc). Recently, it was shown that the neural code for optimal information transmission is maximized for short and sparse bursts^27^. In a subset of L5tt neurons in our data set (*n* = 7), we find that bursting significantly increased upon touch (Figs. 4e, 5g, h). In this subset of L5tt neurons, the predominant fraction of bursts was short (two APs 71%, three APs 19%, four APs 6%, five APs 4%, ≥6 APs 0.5%). Furthermore, we show that in a subset of L5tts, these short burst events carry information on naive whisker touch, which is not present in spike counts (Fig. 6o). Thus, we find sparse, short, and high-information content bursts in S1 during naive, exploratory touch, supporting the hypothesis of a multiplexed neural code^27^.

We were able to use a receiver operating characteristic model to decode whether a touch occurred and expect that decoding touch from spikes and bursts will improve when multiple L5tt neurons are recorded in parallel. Extracellular (silicon probe) recordings can be readily applied in behaving rodents, but clustering methods to reach single-unit resolution assume stable waveforms for individual units and this assumption is violated when L5tt spike waveforms adapt considerably during high-frequency bursts^50^.

In general, the modulation depth of spiking activity during whisking is low, suggesting that meaningful information is present on the population level and much less accurate for individual neurons^66^. Upon touch, L5tts increase spiking activity and relay information to various subcortical (premotor) targets^10,11^. A subset of L5tts additionally represents touch by a temporal code (high-frequency bursting), which is informative for coding and decoding of touch events. Indeed, neurons in POm are driven by L5tt and have the ability to precisely encode descending high-frequency (>100 Hz) cortical information^67^. Remarkably, the information content provided by high-frequency bursts is present in L5tts projecting across multiple, yet segregated subcortical projections (POm, SC, Sp5C, and Pons, Fig. 7). This uncovers the temporal-spatial map of neuronal activity for individual cortical cell types upon sensory-guided behavior.

Tactile processing of whisker information in combination with single-unit recordings in awake conditions is typically studied when rodents are head-fixed and conditioned to a particular task. This can be an object detection task^63^, a (delayed) object discrimination task^68^, or perceptual detection of a passive whisker stimulus^69^. The strategy of behavioral conditioning has several advantages including the display of preferred tactile behavior and the preferred behavior can be triggered by a sensory cue (such as a tone) to focus data acquisition around small temporal epochs (a few seconds for individual trials). Potential disadvantages of the behavioral conditioning approach include a bias of natural behavior towards conditioned behavior and the association of conditioned behavior to action outcome^70,71^. Here, we studied sensory processing in naive rodents that were habituated to head-fixation, but were not trained to perform a task or were not food/water-deprived to increase motivation and action-reward calculations. Voluntary, self-induced tactile behavior was very similar compared with freely moving rats and spiking rates observed across cell types and layers thus highly representative for natural conditions. Our data from untrained, naive rats now provides a stepping stone to compare the coding characteristics during task performance after sensory learning to reveal the neurophysiological correlates in S1 and quantify spike and burst event rates that signal sensory learning, motivation, and reward-expectation^72,73^.

## Methods

All experiments were carried out in accordance with the animal welfare guidelines of the VU Amsterdam, the Netherlands. Male Wistar rats were used (*n* = 48, Charles River, mean postnatal day 39.1 ± 4.1, mean bodyweight 134.9 ± 23.1 grams). Rats were positioned in the recording set-up using a head-post. During surgical preparation, rats were anesthetized using 1.6% isoflurane in 0.4 l/h O_2_ + 0.7 l/h NO_2_ and depth of anesthesia was assured by the absence of foot and eyelid reflexes. In addition, post-operative analgesia using Temgesic (buprenorphine, 0.1–0.5 mg/kg) was given. Body temperature was monitored using a rectal probe and maintained at 37 °C with a heating pad. In the week prior to surgery, rats were handled daily to accustom them to the experimenter and housed in pairs in enriched cages (including shelter, toys, nesting material, food, and water ad libitum). In the week after surgical preparation, rats were head-fixed two times per day for 2–3 days in preparation of the recording session. During the post-surgery week, rats were individually housed in enriched cages with ad libitum food and water and bodyweight was monitored daily. Rats quickly adjusted to the head-fixation period, allowing stable recording configurations without the need for body restraint.

On the recording day, rats were anaesthetized with isoflurane (1.25% in 0.4 l/h O_2_ + 0.7 l/h NO_2_), and targeted loose-patch recordings were made using intrinsic optical imaging^24^. Passive stimulation and receptive field mapping were used to confirm intrinsic optical imaging results and consisted of single whisker deflection at 3.3° in the caudal direction^24^. Afterwards, whiskers were clipped to 5 mm, except the principal or a single surround whisker and anesthesia was terminated. Rats woke up from isoflurane anesthesia within several minutes and spiking during active object touch was quantified only after rats were fully awake, monitored by body posture and exploratory whisking (see also ref. ^74^). We did not find a significant increase in spiking between the start of the awake recordings (20 min after isoflurane termination) and the consecutive 12 min recording (*n* = 10, median ± 1st/3rd quartile, start 1.91 ± 0.74/3.57 Hz, end 1.79 ± 1.18/4.93, Wilcoxon-matched pairs, *p* = 0.82). Thus, once the rats recover from anesthesia and initiate whisker self-motion, we find stable spiking rates across a long temporal window (0–720 s). Active touch resulted from whisker self-motion and was monitored with high-speed videography (MotionScope M3 camera, IDT Europe, Belgium). As rats were not trained to perform tactile behavior and behavior was not rewarded, most of the recording time consisted of quiescent episodes, interleaved with whisking bouts. To allow long continuous recordings, the camera was set to 200 frames/s as trade-off between high temporal resolution and long video segments (375 s continuously at 200 frames/s). Whisker angle was tracked offline^75^ and episodes of whisker movements were classified by applying a fixed threshold across recordings to the power of whisker angle versus time (1–20 Hz bandpass) using the Matlab spectrogram function. The object was positioned 2 cm lateral from the whisker pad and anterior relative to the whisker set point (obtained during quiescent episodes). This ensured that touches were the consequence of whisker protraction. In addition, the proximal position with respect to the whisker follicle ensured that rats would not generate “slip-off” events that can occur with distal object positions. Many touches involved very subtle movements of the whisker with curvature changes below detection threshold. Even for relatively robust touches, signal-to-noise for whisker bending was small, which is a consequence of the proximal position of the object in combination with intrinsic properties of the whisker at the base. Curvature changes upon touch are therefore not included in the analysis. Touch events were detected manually frame-by-frame to achieve accurate time stamps of events resulting from small whisker movements. Behavioral episodes were thus categorized as Q(uiet), W(hisking), or T(ouch).

Loose-patch recordings were made^35^ and individual neurons recorded using patch pipettes (5–7 MΩ) filled with (in mM): 135 NaCl, 5.4 KCl, 1.8 CaCl_2_, 1 MgCl_2_, 5 Hepes, 2% biocytin, pH adjusted to 7.2 with NaOH. To ensure unbiased sampling (irrespective of spiking frequency), single neurons were searched for by monitoring electrode resistance while lowering the electrode in 1 µm steps. Neurons were filled with biocytin using electroporation after the acquisition of functional characteristics. Fast-spiking units (*n* = 13) were categorized based on AP waveform in combination with morphology and recording depth^44^. Data exclusively represents stable, somatic recordings with unprecedented single-unit isolation. Morphology after post hoc histology allowed cell-type classification for the major fraction of regular spiking units (48 out of 67 units, examples in Fig. 2). RSU recordings in layers L2-4 and L6 without recovered morphology (*n* = 12) were assigned to L2/3, L4, and L6 based on loose-patch depth measurements^39^ in combination with established layer borders^76^. In L5, 23 out of 30 RSUs were classified on morphological characteristics. The remaining *n* = 7 units were classified as putative L5tt neurons based on short-latency responses to mechanical whisker deflection, broad receptive fields, and high spontaneous spiking under anesthesia, which represent functional properties that distinguish L5tt neurons from L5st neurons under anesthesia^24,41^. L5 recordings were not included in the analysis when morphology was missing and in case of ambiguous physiological signature. Due to limited sample sizes, we did not further subclassify cell types in L4 or L6.

After in vivo experiments, rats were deeply anaesthetized with urethane (>2.0 g/kg) and perfused with 0.9% NaCl, then 4% paraformaldehyde (PFA). Brains were post-fixed in 4% PFA overnight at 4 °C and transferred to 0.9% NaCl. Tangential sections (100 µm) were cut on a vibratome and stained using a modified avidin-biotin peroxidase method.

### Data analysis

High-frequency burst event: defined as a set of spikes with consecutive ISI of ≤10 ms, (instantaneous spiking frequency of ≥100 Hz), which is the critical frequency for dendritic calcium electrogenesis in distal compartments of neurons in the primary somatosensory cortex, which in turn determines dendritic plasticity mechanisms^45^. Burst rate (Fig. 4d, e) is calculated by dividing the number of burst events by the total time for a particular behavioral category (event/s). Occurrence of burst length of different sizes (Fig. 4f, g) is calculated as the fraction of bursts consisting of 2, 3, 4, 5, or ≥6 spikes with all intraburst ISI ≤ 10 ms, relative to all burst events.

Decoding analysis: spike trains were binned within a 100 ms sliding window with 10 ms steps. Each 100 ms bin was matched to our previously identified behavioral episodes: “Quiescent” comprises bins completely embedded within Quiet episodes. “Touch” bins comprised windows within 50 ms before touch onset and 150 ms after touch onset (i.e., ranging from −50 to 50 ms and +50 to +150 ms relative to touch onset). Bins only partially within an episode, bins within non-touch whisking, and bins within touch episodes but later than 150 ms after touch onset were not incorporated in this analysis. For each bin, we determined the spike count and the burst event count. The decoding analysis was performed separately on spike count and burst spiking. We obtained the distribution of spike counts for all windows during touch epochs (T distribution) as well as for all windows during quiescent epochs (Q distribution). We built an ROC curve by varying a classification criterion along with all spike count values and computing the true-positive rate (the proportion of windows from the T distribution, properly classified as touch) and false-positive rate (the proportion of windows from the Q distribution, wrongly classified as touch). Decoding performance was estimated as the AUROC, a metric that represents how well an ideal observer can decode touch versus no-touch based solely on the given neuron’s spiking rate^77–79^. The AUROC was computed using MATLAB’s perfcurve function. Neurons with increased spiking (or bursting) upon touch result in an AUROC > 0.5; neurons with decreased spiking (or bursting) upon touch result in an AUROC < 0.5. Finally, absolute decoding power was calculated as the deviation from chance level (AUROC = 0.5), independent of increased/decreased spiking (or bursting). Statistical significance was determined using a permutation test: for each neuron, we randomly shuffled the touch labels 5000 times and re-computed decoding performance to create the random-chance decoding performance distribution (H0), used to compute the observed decoding performance *p* value. Individual neurons with a decoding performance with a *p* value < 0.01 after correction for false discovery rate (Benjamini–Hochberg procedure) were considered to hold significant decoding power. The same analysis was used to determine decoding power in (1) spike rate or (2) burst events.

### Jensen–shannon divergence

The Jensen-Shannon divergence (JSD) is an information-theoretic measure of similarity between probability distributions. Its square root (√JSD) is a true distance metric called Jensen–Shannon Distance^47^. We used it to measure how well one can separate Quiescent from Touch distributions using the available information (Spike count and Burst presence). We used the same bins from the decoding analysis to obtain, for Quiescent and Touch bins separately, an empirical joint probability mass function with two dimensions: spike count (*S*) and burst presence (*B*).

$$\forall s\,\,{\mathbb{\in }}\,\,{\mathbb{N}}{\mathbb{,}}{\mathbb{\forall }}b\in \left[0,1\right]{\rm{:}}$$

$${T=P}_{\rm{TOUCH}}\left(S=s,B=b\right)=\,\frac{{\rm{\#}}{{{\rm{Touch}}\;{\rm{bins}}\;{\rm{with}}\;{\rm{S}}}}={{{\rm{s}}\;{\rm{and}}\;{\rm{B}}}}={\rm{b}}}{{{\#{\rm{Touch}}\;{\rm{bins}}}}}$$

$${Q=P}_{\rm{QUIESCENT}}\left(S=s,B=b\right)=\frac{{{\#}}{{{\rm{Quiescent}}\;{\rm{bins}}\;{\rm{with}}\;{\rm{S}}}}={{{\rm{s}}\;{\rm{and}}\;{\rm{B}}}}={\rm{b}}}{{{\#{\rm{Quiescent}}\;{\rm{bins}}}}}$$

We next computed the JSD between these two probability distributions as:

$${JSD}(T||Q)=H\left(\frac{T+Q}{2}\right)-\frac{H\left(T\right)+H(Q)}{2}$$

Where H is Shannon entropy^80,81^. We used bootstrapping to assess the variability of the measured √JSD: for each cell, we re-sampled the data by constructing a new vector of the same size but filled with data points randomly sampled with replacement from the original data. We repeated this 1000 times and re-computed √JSD. We used the 2.5th and 97.5th percentiles of the bootstrapped √JSD distribution as the boundaries of the 95% confidence interval.

Next, in order to assess the separability of *P*_Quiescent_ and *P*_Touch_ distributions that is solely due to burst presence or absence (i.e., excluding information gained from spike count), we computed their √JSD when shuffling the burst labels 8000 times. We used the difference between the observed √JSD value and the mean of the √JSD burst-shuffled distribution as a measure of separability gained by bursts alone. *P* values were computed from the shuffle distribution and the significance threshold was set at *p* < 0.01 after correction for false discovery rate (Benjamini–Hochberg procedure).

### Statistics and reproducibility

Statistical analyses were made using Graphpad InStat 3 (GraphPad Software, Inc, La Jolla, USA) and Matlab R2009b, 2013 or 2017b using custom-written software (Mathworks, Natick, USA). Non-parametric data were visualized in boxplots (generated in Matlab 2017b) with the central mark as the median, the edges of the box the 25th and 75th percentiles, the whiskers extend to the most extreme data points, and the outliers are plotted individually. Data were collected within the framework of this project and we did not specifically attempt to replicate the current data set.

### Reporting summary

Further information on research design is available in the Nature Research Reporting Summary linked to this article.

## Supplementary information

Reporting Summary

## Data Availability

The data sets generated during and/or analyzed during the current study are available from the corresponding author on reasonable request.

## Code availability

The codes used during the current study are available from the corresponding author on reasonable request.
