# Supplementary material for: High-frequency burst spiking in layer 5 thick-tufted pyramids of rat primary somatosensory cortex encodes exploratory touch
Source: Commun Biol. 2021 Jun 10;4:709. doi: 10.1038/s42003-021-02241-8 (PMC8192911; doi:10.1038/s42003-021-02241-8)
Supplement: Supplementary file 1 — Reporting Summary [file 42003_2021_2241_MOESM1_ESM.pdf]

nature research

Corresponding author(s): CPJ de Kock

Last updated by author(s): May 6, 2021

Reporting Summary

Nature Research wishes to improve the reproducibility of the work that we publish. This form provides structure for consistency and transparency in reporting. For further information on Nature Research policies, see our Editorial Policies and the Editorial Policy Checklist.

Statistics

For all statistical analyses, confirm that the following items are present in the figure legend, table legend, main text, or Methods section.

n/a	Confirmed	
☐	☒	The exact sample size (*n*) for each experimental group/condition, given as a discrete number and unit of measurement
☐	☒	A statement on whether measurements were taken from distinct samples or whether the same sample was measured repeatedly
☐	☒	The statistical test(s) used AND whether they are one- or two-sided<br>*Only common tests should be described solely by name; describe more complex techniques in the Methods section.*
☐	☒	A description of all covariates tested
☐	☒	A description of any assumptions or corrections, such as tests of normality and adjustment for multiple comparisons
☐	☒	A full description of the statistical parameters including central tendency (e.g. means) or other basic estimates (e.g. regression coefficient) AND variation (e.g. standard deviation) or associated estimates of uncertainty (e.g. confidence intervals)
☐	☒	For null hypothesis testing, the test statistic (e.g. *F*, *t*, *r*) with confidence intervals, effect sizes, degrees of freedom and *P* value noted<br>*Give P values as exact values whenever suitable.*
☒	☐	For Bayesian analysis, information on the choice of priors and Markov chain Monte Carlo settings
☐	☒	For hierarchical and complex designs, identification of the appropriate level for tests and full reporting of outcomes
☐	☒	Estimates of effect sizes (e.g. Cohen's *d*, Pearson's *r*), indicating how they were calculated

Our web collection on statistics for biologists contains articles on many of the points above.

Software and code

Policy information about availability of computer code

Data collection	Electrophysiology: Recordings were made using an Axoclamp 2B amplifier (Axon instruments,UnionCity, CA, USA) in combination with a Lynx 8 amplifier, band filter settings 300 and 9000 Hz. Data were acquired using the Ntrode Virtual Instrument (R. Bruno,Columbia Univ, NY) for Labview (National Instruments, Austin, TX, USA).<br>Behavior: whisker self-motion was monitored with high-speed videography (MotionScope M3 camera, IDT Europe, Belgium)
Data analysis	Electrophysiological data was analyzed using custom written software in Matlab (Mathworks, Natick, USA). Behavioral data was analyzed off-line using tracking software (Knutsen et al, JNeurophysiol 2005).

For manuscripts utilizing custom algorithms or software that are central to the research but not yet described in published literature, software must be made available to editors and reviewers. We strongly encourage code deposition in a community repository (e.g. GitHub). See the Nature Research guidelines for submitting code & software for further information.

Data

Policy information about availability of data

All manuscripts must include a data availability statement. This statement should provide the following information, where applicable:

- Accession codes, unique identifiers, or web links for publicly available datasets
- A list of figures that have associated raw data
- A description of any restrictions on data availability

The datasets generated during and/or analyzed during the current study are available from the corresponding author on reasonable request.

Field-specific reporting

Please select the one below that is the best fit for your research. If you are not sure, read the appropriate sections before making your selection.

☒ Life sciences ☐ Behavioural & social sciences ☐ Ecological, evolutionary & environmental sciences

For a reference copy of the document with all sections, see nature.com/documents/nr-reporting-summary-flat.pdf

Life sciences study design

All studies must disclose on these points even when the disclosure is negative.

Sample size	Sample-size calculation was not used. Multiple major conclusions are based on single cell properties across morphological types (instead of population means/medians), which make calculations for population sizes less informative. Most important cell-type specific properties for which population properties (bursting, Fig.5) are quantified, include samples of n>10 per individual category, which is the threshold to determine parametric/non-parametric statistics and a valid representation of the underlying distribution.
Data exclusions	Data was excluded when morphological identification was not possible and in case of ambiguous physiological signature.
Replication	Data was collected within the framework of this project. We did not specifically attempt to replicate the current dataset, but put the results in a broad framework in the discussion.
Randomization	Samples were grouped according to cell-type classification parameters.
Blinding	During the recording, the experimenter is blind to the morphological identity of the recorded neuron since the identity can only be determined through post-hoc histology.

Reporting for specific materials, systems and methods

We require information from authors about some types of materials, experimental systems and methods used in many studies. Here, indicate whether each material, system or method listed is relevant to your study. If you are not sure if a list item applies to your research, read the appropriate section before selecting a response.

Materials & experimental systems

n/a	Involved in the study	
☒	☐	Antibodies
☒	☐	Eukaryotic cell lines
☒	☐	Palaeontology and archaeology
☐	☒	Animals and other organisms
☒	☐	Human research participants
☒	☐	Clinical data
☒	☐	Dual use research of concern

Methods

n/a	Involved in the study	
☒	☐	ChIP-seq
☒	☐	Flow cytometry
☒	☐	MRI-based neuroimaging

Animals and other organisms

Policy information about studies involving animals; ARRIVE guidelines recommended for reporting animal research

Laboratory animals	Wistar, rat, males, mean postnatal day 39.1 ± 4.1
Wild animals	*Provide details on animals observed in or captured in the field; report species, sex and age where possible. Describe how animals were caught and transported and what happened to captive animals after the study (if killed, explain why and describe method; if released, say where and when) OR state that the study did not involve wild animals.*
Field-collected samples	*For laboratory work with field-collected samples, describe all relevant parameters such as housing, maintenance, temperature, photoperiod and end-of-experiment protocol OR state that the study did not involve samples collected from the field.*
Ethics oversight	All experiments were carried out in accordance with the animal welfare guidelines of the VU Amsterdam, the Netherlands, licence INF1301-A1.

Note that full information on the approval of the study protocol must also be provided in the manuscript.
